# Identifying novel genes and biological processes relevant to the development of cancer therapy-induced mucositis: An informative gene network analysis

**DOI:** 10.1371/journal.pone.0180396

**Published:** 2017-07-05

**Authors:** Cielito C. Reyes-Gibby, Stephanie C. Melkonian, Jian Wang, Robert K. Yu, Samuel A. Shelburne, Charles Lu, Gary Brandon Gunn, Mark S. Chambers, Ehab Y. Hanna, Sai-Ching J. Yeung, Sanjay Shete

**Affiliations:** 1 Department of Emergency Medicine, The University of Texas MD Anderson Cancer Center, Houston, Texas, United States of America; 2 Department of Biostatistics, The University of Texas MD Anderson Cancer Center, Houston, Texas, United States of America; 3 Department of Infectious Diseases, The University of Texas MD Anderson Cancer Center, Houston, Texas, United States of America; 4 Department of Thoracic Head and Neck Medical Oncology, The University of Texas MD Anderson Cancer Center, Houston, Texas, United States of America; 5 Department of Radiation Oncology, The University of Texas MD Anderson Cancer Center, Houston, Texas, United States of America; 6 Department of Head and Neck Surgery, The University of Texas MD Anderson Cancer Center, Houston, Texas, United States of America; 7 Department of Epidemiology, The University of Texas MD Anderson Cancer Center, Houston, Texas, United States of America; South Texas Veterans Health Care System, UNITED STATES

## Abstract

Mucositis is a complex, dose-limiting toxicity of chemotherapy or radiotherapy that leads to painful mouth ulcers, difficulty eating or swallowing, gastrointestinal distress, and reduced quality of life for patients with cancer. Mucositis is most common for those undergoing high-dose chemotherapy and hematopoietic stem cell transplantation and for those being treated for malignancies of the head and neck. Treatment and management of mucositis remain challenging. It is expected that multiple genes are involved in the formation, severity, and persistence of mucositis. We used Ingenuity Pathway Analysis (IPA), a novel network-based approach that integrates complex intracellular and intercellular interactions involved in diseases, to systematically explore the molecular complexity of mucositis. As a first step, we searched the literature to identify genes that harbor or are close to the genetic variants significantly associated with mucositis. Our literature review identified 27 candidate genes, of which *ERCC1*, *XRCC1*, and *MTHFR* were the most frequently studied for mucositis. On the basis of this 27-gene list, we used IPA to generate gene networks for mucositis. The most biologically significant novel molecules identified through IPA analyses included *TP53*, *CTNNB1*, *MYC*, *RB1*, P38 MAPK, and *EP300*. Additionally, uracil degradation II (reductive) and thymine degradation pathways (*p* = 1.06^−08^) were most significant. Finally, utilizing 66 SNPs within the 8 most connected IPA-derived candidate molecules, we conducted a genetic association study for oral mucositis in the head and neck cancer patients who were treated using chemotherapy and/or radiation therapy (186 head and neck cancer patients with oral mucositis vs. 699 head and neck cancer patients without oral mucositis). The top ranked gene identified through this association analysis was *RB1* (rs2227311, *p*-value = 0.034, odds ratio = 0.67). In conclusion, gene network analysis identified novel molecules and biological processes, including pathways related to inflammation and oxidative stress, that are relevant to mucositis development, thus providing the basis for future studies to improve the management and treatment of mucositis in patients with cancer.

## Introduction

Mucositis is a toxicity (inflammation/ulceration) of the alimentary tract resulting from chemotherapeutic agents or radiation [1–3]. Mucositis represents a major complication for patients undergoing cancer treatment [4, 5]: oral and gastrointestinal mucositis are most commonly found in nearly 100% of patients undergoing high-dose chemotherapy and hematopoietic stem cell transplantation [3] and in 60%–80% of patients being treated for malignancies of the head and neck [6]. Complications of mucositis include painful mouth ulcers, difficulty eating or swallowing, and gastrointestinal distress (including diarrhea), which can result in unplanned hospitalizations, poorer clinical outcomes, and reduced quality of life [2, 7]. Current treatment focuses on pain control, rehydration, and basic oral/bowel care, but these have not been shown to meaningfully influence the trajectory of mucositis [4]. Clinical factors such as dose, duration, and intensity of radiation and chemotherapy [4], and patient factors such as age, sex, and body mass index [7–10], have been found to be associated with the development of mucositis, yet these explain only some of the variation observed in the risk for, severity of, and persistence of this condition. Therefore, a better understanding of the potential biological mechanisms underlying mucositis development, severity, and persistence may have significant clinical impact.

Technological advances in molecular biology have allowed for a better understanding of cancer treatment-related toxicities. For example, candidate gene studies of mucositis have found genetic variants or single-nucleotide polymorphisms (SNPs), i.e., DNA damage repair and apoptosis, that potentially underlie the development of mucositis related to cancer treatment. However, these genetic associations remain poorly characterized [11–15], with the literature suggesting that the pathogenesis of mucositis is much more complex [7] (see Table 1). More recently, network-based approaches have enabled researchers to integrate the complex intracellular and intercellular interactions involved in disease, but these approaches have not been used to systematically explore the molecular complexity of mucositis. Therefore, in this study, we used Ingenuity Pathway Analysis (IPA; Ingenuity^®^ Systems, www.ingenuity.com), a bioinformatic tool, to explore biological pathways and molecular mechanisms associated with mucositis. Finally, using the IPA-derived candidate molecules, we conducted a genetic association study for oral mucositis in the head and neck cancer patients who were treated using chemotherapy and/or radiation therapy. The overall goal was to provide a better understanding of potential biological markers of mucositis as a means to develop novel investigative and treatment approaches.

**Table 1:** Five biological stages of mucositis [2, 3].

Initiation	DNA damage (reversible and irreversible)
Primary damage response	Pathways triggered by DNA strand breaks and lipid transduction pathways prompt activation of transcription factors, including nuclear factor kappa B, p53, and associated pathways
Signaling and amplification	Apoptosis and tissue injury
Ulceration	Damage and apoptotic changes to mucosal epithelium
Healing	Ulcer resolution: the submucosa's extracellular matrix guides proliferation, migration, and differentiation of the epithelium bordering the ulcer

## Materials and methods

We first conducted a literature search on genetic studies of mucositis, as described below. Second, using genes pooled from the literature as a starting point, we used IPA to generate gene networks for mucositis and identified additional molecules that are functionally related to the genes obtained from the literature search.

### Literature search

We performed a comprehensive literature review through the PubMed database. The search was limited to human studies and articles published in English prior to April 2016. We aimed to identify genes that harbor or are close to genetic variants associated with mucositis in cancer patients, which would then serve as “focus genes” in the IPA described next. We used “SNP(s) and mucositis” and “gene(s) and mucositis” as search terms. Singular and plural keywords were used separately for the literature search, as we anticipated that each search could identify additional studies.

Initially, we screened articles on the basis of the title, abstract, and full text. Duplicate articles, non-human trials, literature reviews, clinical trials, studies of unrelated phenotypes, and meta-analyses were excluded. We subsequently manually searched the reference lists of the selected articles and of related review articles to identify additional relevant studies for inclusion. From the selected studies, we retrieved information about genes that harbor or are close to significantly associated genetic variants (SNPs or haplotypes), as reported in the articles, and included those genes in the IPA. The literature search and information extraction were conducted by Stephanie C. Melkonian in April 2016.

### Ingenuity Pathway Analysis

We used IPA to produce and connect a comprehensive list of molecules potentially associated with the development of mucositis in cancer patients. IPA is a bioinformatic tool that connects a list of molecules into a set of networks based on the Ingenuity Knowledge Base, which contains information on biomolecules (represented by nodes in the networks) and their relationships (represented by edges and arrows in the networks) [16].

On the basis of the focus genes identified through the literature search, we used the core analysis function in IPA to identify biological functions, signaling and metabolic pathways, as well as molecular networks, which include the molecules potentially directly or indirectly associated with mucositis in cancer patients. In IPA core analysis, each focus gene is weighted equally, regardless of how many times it is reported in the literature. To generate the molecular network, a key assumption is that the biological function of a network involves locally dense interactions [17–20]. On the basis of this assumption, the network-generating process in IPA uses the triangular connectivity between molecules (i.e., the number of pairs of molecules to which a molecules is connected), which favors denser networks over relatively sparse ones [17]. The network-generating process has been described in detail previously [21, 22]. Briefly, this process includes ranking the focus genes using the triangular connectivity and generating seed gene networks using focus genes. The process is repeated until all the focus genes are represented in a relevant network [17]. If the resulting network does not reach the maximum network size pre-specified by IPA, that is, 35, 70 or 140 molecules per network, additional molecules or networks are connected to the existing networks from IPA’s database. In the current study, we used 140 molecules per network as the maximum network size since a larger network increases the chance of including all focus genes in the same network [23]. When adding molecules or networks from the database, IPA uses the specific connectivity metric, which gives priority to molecules that have the largest overlap with the existing network and have the lowest number of neighbors. A focus gene can be excluded from the network if it is less likely to have connections (biological relationships) with the network.

The resulting pathways, functions and networks are scored on the basis of the negative base-10 logarithm of the *p*-value from a right-tailed Fisher’s exact test. The *p*-values obtained using this test identify statistically significant enrichment of the focus genes in a given function, pathway, or network [24]. In addition to the nominal *p*-values, we also report the Benjamini-Hochberg multiple testing correction *p*-values (B-H *p*-values) for canonical pathways and biological functions. The Benjamini-Hochberg method corrects *p*-values to account for multiple testing [25]. We used a significance level of < 10^−5^ (score > 5) to select networks.

### Mucositis genetic association in head and neck cancer patients

We conducted the genetic association study for oral mucositis in head and neck cancer patients using the most interconnected molecules identified from the IPA core analysis as the candidate molecules. We used the genetic data available for the adult head and neck cancer patients who have been treated previously with chemotherapy and/or radiation therapy. All patients provided written informed consent and the study was approved by the Institutional Review Board at MD Anderson Cancer Center. Oral mucositis was diagnosed based on both ICD9 codes (528, 528.01, 528.02) and ICD10 codes (K12.30, K12.32, K12.33). The study population included 885 head and neck cancer patients, of which 186 patients had oral mucositis and 699 patients did not have oral mucositis. There were 41 female and 145 male patients represented in the oral mucositis cases (mean age 58 years, standard deviation [sd] = 9); and 126 female and 573 male patients in the controls (mean age 57 years, sd = 11). Genotyping for all patients was conducted at MD Anderson Cancer Center with the use of the Illumina HumanOmniExpress-12v1 BeadChip.

Statistical analyses were conducted using PLINK [26] and R program. For quality control purposes, we used the 1 degree-of-freedom *χ*^*2*^ test or Fisher’s exact test to assess the deviation from the Hardy-Weinberg proportion (HWP). SNPs with minor allele frequencies (MAFs) ≤ 5% and HWP *p*-values ≤ 10^−6^ were excluded from the analyses. The association between each SNP and oral mucositis status was assessed using multivariable unconditional logistic regression, adjusting for sex, age, study batches and cluster information. The clusters of patients were identified by using a nearest neighbor cluster analysis based on genetic similarity. For each IPA-derived molecule, we report the SNP with the lowest *p*-value belonging to it (a gene or a group of genes) [27–31].

## Results

### Literature review

**Fig 1:**
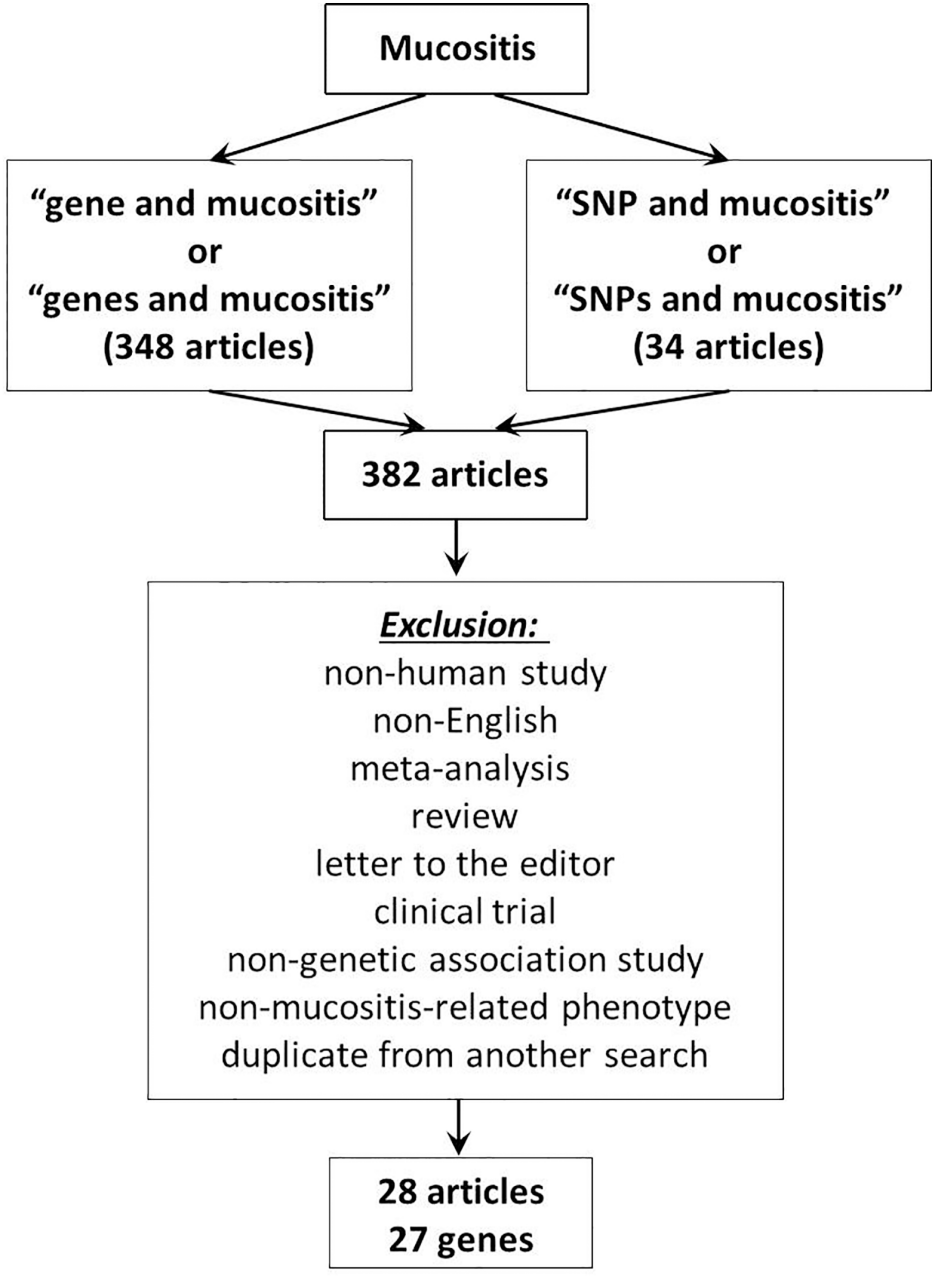
Literature search flow chart. *Exclusion criteria: (1) not a human study, (2) not published in English, (3) meta-analysis, review, or letter to the editor, (4) clinical trial, (5) not a genetic association study, (6) not a mucositis-related phenotype study, or (7) duplicate from another search.

The overall flow chart of the literature review is shown in Fig 1. From our search of different terms in the PubMed database, we identified a total of 382 articles. After screening the title, abstract and full text, we excluded articles for the following reasons: (1) not a human study, (2) not published in English, (3) meta-analysis study, review, or letter to the editor, (4) clinical trial, (5) not a genetic association study, (6) not a mucositis-related phenotype study, or (7) duplicate from another search. After these exclusions, 28 articles remained from our search; from these we identified 27 genes, which served as our focus genes in the IPA.

Table 2 lists the year of publication, first author, patient ethnicity, cancer type, sample size, sample source, phenotype, and significant genes from the articles in our literature search. These studies included various cancer sites and multiple ethnicities. Whereas all studies included mucositis as the phenotype of interest, several of the studies included other cancer treatment-related toxicities [32–35]. Several overlapping focus genes were observed across the various studies, with the most commonly cited genes being *ERCC1*, *XRCC1*, and *MTHFR*.

**Table 2:** Summary of literature search.

Year	First Author	Ethnicity	Cancer Type	Sample Size	Sample	Phenotype	Significant Genes
2015	den Hoed [36]	C	LL	134	Peripheral blood	Mucositis	*ABCC4*
2015	Teo [37]	A	RCC		Blood	Mucositis	*ABCB1*
2015	Coleman [38]	C	MM	972	MIRT bank of germline DNA from leukapheresis products	Mucositis	*JPH3*, *DHRS7C*, *CEP192*, *CPEB1/LINC00692*, *FBN2*, *ALDH1A1*, *DMRTA1/FLJ35282*
2014	Boso [39]	C	Breast cancer	113	Blood	Toxicity (mucositis)	*ERCC1*
2014	Venkatesh [14]	C	Head and neck	183	Not specified	Acute toxicity (mucositis) > Grade 2	*NBN*, *XRCC1*
2014	Liu [40]	A	ALL	112	Peripheral blood	Nonhematologic toxicity (mucositis)	*ABCC2*
2014	Ren [13]	C	Nasopharyngeal carcinoma	120	Peripheral blood	Severe oral mucositis	*XRCC6*
2013	Yomade [41]	C	ALL	131	Peripheral blood	Mucositis	*NOD2*
2012	Dogan [34]	C	Various	18 (children)	Peripheral blood	Clinical/biochemical toxicity (mucositis)	*DPYD*, *TYMS*, *MTHFR*, *XPD*, *XRCC1*
2012	Bektas-Kayhan [42]	C	ALL	47 cases/68 controls	Blood	Mucositis	*MDR*
2012	Fidlerova [43]	C	Various	113	Blood	Mucositis	*UPB1*
2012	Chen [44]	A	AL	96 (AL) and 132 (controls) (children)	Bone marrow (AL) Peripheral blood (controls)	Mucositis	*GGH*
2012	Ozdemir [12]	C	Burkitt lymphoma/ALL	90 (children)	Venous blood	Mucositis	*XRCC1*
2012	Erculj [35]	C	ALL	167 (children)	Peripheral blood	Mucositis	*TYMS*
2011	Thomas [33]	C	Rectal cancer	131	Blood	Toxicity (mucositis)	*MTHFR*
2011	Pratesi [45]	C	HNSCC	101	Peripheral blood	Severe oral mucositis	*XRCC1*
2011	Kotnik [46]	C	ALL/lymphoma	64 (children)	Peripheral blood	Mucositis	*TYMS*
2010	Banklau [47]	A	ALL/LL	94	Peripheral blood	Mucositis	*DCK*
2010	Cho [48]	A	Large B cell lymphoma	94	Peripheral blood	Mucositis	*GSTT1*
2010	Dumontet [49]	C	MM	169	Peripheral blood	Severe mucositis	*BRCA1*, *CDKN1A*, *XRCC1*
2010	Tantawy [50]	C	LL	40	Peripheral blood	Mucositis/mucosal toxicity	*MTHFR*
2010	Fidlerova [51]	C	Various	113	Blood	Mucositis	*DPYS*
2009	van Erp [52]	C	Various	219	Blood or serum	Mucosal inflammation	*CYP1A1*
2009	Kleibl [53]	C	Colorectal	76	Blood	Mucositis	*DPYD*
2009	Rocha [32]	C	Leukemia	107 patients/ 107 donors	Peripheral blood	Mucositis	*CYP2B6*
2008	Schwab [54]	C	Various	683	Peripheral blood	Toxicity (severe leukopenia, diarrhea and mucositis)	*DPYD*
2007	Gemmati [55]	C	NHL	110	Peripheral blood	Mucositis	*MTHFR*
2006	Robien [56]	C	CML	172	Not specified	Mucositis	*MTHFR*
2001	Ulrich [57]	C	CML	220	Not specified	Mucositis (oral mucositis index)	*MTHFR*

Ethnicity: C, Caucasian; A, Asian. Cancer type: LL, lymphoblastic lymphoma; RCC, renal cell carcinoma; MM, multiple myeloma; AL, acute leukemia; ALL, acute lymphoblastic leukemia; HNSCC, squamous cell carcinoma of the head and neck; NHL, non-Hodgkin lymphoma; CML, chronic myeloid leukemia.

### IPA core analysis

**Fig 2:**
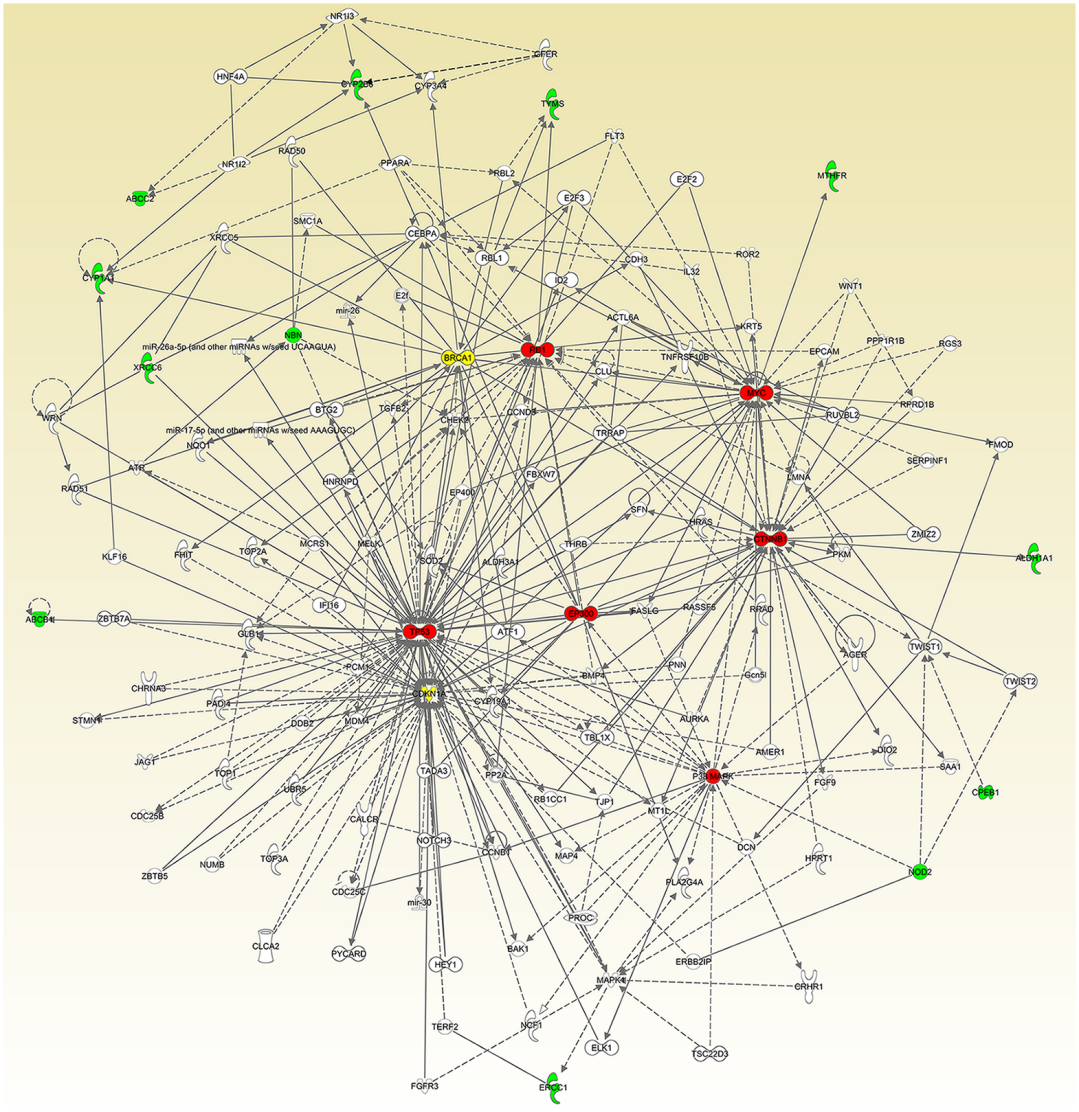
Most significant network (*p* = 10^−22^) generated by IPA core analysis for mucositis, using 27 focus genes. Green nodes: focus genes with fewer than 15 connections; red nodes: non-focus molecules with at least 15 connections; yellow nodes: focus genes with at least 15 connections. Dashed and solid lines represent indirect and direct interactions, respectively.

We performed the IPA core analysis for the focus genes reported to be associated with treatment-induced mucositis. The significant network (*p* = 10^−22^) revealed from the IPA core analysis is shown in Fig 2. In this network, the solid and dashed edges or arrows indicate direct and indirect interactions, respectively. The green nodes indicate focus genes (identified via the literature review) with fewer than 15 connections, the red nodes indicate molecules, which are not focus genes, with at least 15 connections, and the yellow nodes indicate focus genes with at least 15 connections. The figure for each of the molecules with at least 15 connections and the molecules connected to it were provided in the Supporting Information S1 Fig.

We were particularly interested in the molecules with the most interconnections since it has been hypothesized that highly connected molecules (called hubs) are most likely associated with diseases or biological functions [23, 58–60]. There is no consensus in the literature on the cutoff threshold to use in defining hubs in a biological network [61]. Researchers used different criteria to define hubs, such as defining the top 95% and 50% of the high-degree nodes as hubs in different contexts [62] or defining the nodes with degrees greater than 5 [63], 8 [64] or 20 [65] as hubs in different studies. Here, the degree of a node is the number of nodes with which it interconnects [64]. In our study, we considered molecules with degrees (i.e., number of connections) greater than or equal to 15 as hubs. Table 3 shows the molecules that had at least 15 connections (suggesting biological importance) in the networks, ranked by the number of connections for each listed molecule. The focus genes with at least 15 connections were *CDKN1A* and *BRCA1*; the molecules identified through the IPA were *TP53*, *CTNNB1*, *MYC*, *RB1*, P38 MAPK, and *EP300*. We also listed the focus genes with <15 connections in the network in Table 3.

**Table 3:** Molecules with ≥ 15 connections (hubs*) and focus genes with < 15 connections in the network, ranked by number of connections.

IPA symbol	Focus gene	Molecule type	Number of connections
*TP53*	No	transcription regulator	88
*CDKN1A*	Yes	kinase	51
*CTNNB1*	No	transcription regulator	39
*MYC*	No	transcription regulator	36
*RB1*	No	transcription regulator	26
P38 MAPK	No	group	22
*BRCA1*	Yes	transcription regulator	20
*EP300*	No	transcription regulator	16
*NBN*	Yes	other	6
*XRCC6*	Yes	enzyme	6
*CYP2B6*	Yes	enzyme	5
*NOD2*	Yes	other	4
*CYP1A1*	Yes	enzyme	3
*TYMS*	Yes	enzyme	3
*ABCC2*	Yes	transporter	2
*ERCC1*	Yes	enzyme	2
*ABCB1*	Yes	transporter	1
*ALDH1A1*	Yes	enzyme	1
*CPEB1*	Yes	translation regulator	1
*MTHFR*	Yes	enzyme	1

* Suggests biological importance

### Top canonical pathways and diseases and functions

In addition to identifying the network, the IPA core analysis provided the most significant canonical pathways and biological functions (Tables 4 and 5). Table 4 shows the top 6 canonical pathways with B-H *p*-value < 10^−5^ discovered by the IPA core analysis of the focus genes reported in the literature as having associations with cancer treatment-related mucositis. Additionally, 19 canonical pathways of potential interest are listed in the Supporting Information S1 Table. The significant *p*-value in this analysis implies overrepresentation of focus genes in that particular pathway. The canonical pathways represented in Table 4 and S1 Table are ranked in order by B-H *p*-value. Table 4 also lists the number of molecules in the canonical pathways, number of focus genes involved in the canonical pathways, and ratio of the number of focus genes that are included in the canonical pathway to the number of the molecules that make up the canonical pathway listed. In this analysis, the two most significant pathways were the uracil degradation II (reductive) and thymine degradation pathways (B-H *p* = 4.02^−7^). The canonical pathway with the highest ratio was glutamate removal from folates (100%) (S1 Table).

**Table 4:** Top canonical pathways (B-H *p*-value^Ɨ^ < 10^−5^) discovered by IPA core analysis*.

Ingenuity Canonical Pathways	*p*-value	B-H *p*-value	# of molecules in pathway	# of focus genes	Ratio^ǂ^
Uracil Degradation II (Reductive)	1.06E-08	4.02E-07	4	3	75%
Thymine Degradation	1.06E-08	4.02E-07	4	3	75%
LPS/IL-1 Mediated Inhibition of RXR Function	4.18E-07	1.06E-05	208	6	3%
DNA Double-Strand Break Repair by Non-Homologous End Joining	9.53E-07	1.81E-05	14	3	21%
Xenobiotic Metabolism Signaling	1.40E-06	2.14E-05	256	6	2%
PXR/RXR Activation	1.90E-06	2.41E-05	63	4	6%

* Ranked by B-H *p*-value.

^Ɨ^ Benjamini-Hochberg multiple testing correction *p*-value.

^ǂ^ Ratios are calculated by taking the number of focus genes that are included in the canonical pathway divided by the number of the molecules that make up the canonical pathway.

**Table 5:** Top 25 diseases and functions discovered by IPA core analysis of focus genes*.

Categories	Functions	*p*-value	B-H *p*-value^Ɨ^
Cancer, Organismal Injury and Abnormalities, Skeletal and Muscular Disorders	myosarcoma	4.27E-09	1.59E-06
Cancer, Organismal Injury and Abnormalities, Skeletal and Muscular Disorders	muscle tumor	5.37E-09	1.59E-06
Organismal Survival	survival of organism	6.25E-09	1.59E-06
Cancer, Organismal Injury and Abnormalities, Skeletal and Muscular Disorders	rhabdomyosarcoma	4.37E-08	8.31E-06
Cancer, Organismal Injury and Abnormalities, Skeletal and Muscular Disorders	embryonal rhabdomyosarcoma	8.47E-08	1.29E-05
Cancer, Organismal Injury and Abnormalities, Respiratory Disease	lung tumor	1.25E-07	1.59E-05
Cell Cycle	G2/M phase	3.14E-07	3.41E-05
Cancer, Cardiovascular Disease, Organismal Injury and Abnormalities	hemangioblastoma	5.40E-07	5.13E-05
DNA Replication, Recombination, and Repair	repair of DNA	7.72E-07	6.53E-05
Gastrointestinal Disease, Inflammatory Disease	mucositis	9.53E-07	7.25E-05
Cancer, Organismal Injury and Abnormalities, Reproductive System Disease	breast cancer	1.11E-06	7.68E-05
Connective Tissue Disorders, Skeletal and Muscular Disorders	arthropathy	1.35E-06	8.58E-05
Cancer, Hereditary Disorder, Organismal Injury and Abnormalities	susceptibility to familial breast-ovarian cancer type 1	1.99E-06	1.16E-04
Cancer, Organismal Injury and Abnormalities	precancerous condition	2.74E-06	1.49E-04
Cancer, Organismal Injury and Abnormalities	breast or ovarian cancer	3.26E-06	1.65E-04
Cancer, Organismal Injury and Abnormalities, Reproductive System Disease	HER2 negative hormone receptor negative breast cancer	5.63E-06	2.52E-04
Nucleic Acid Metabolism, Small Molecule Biochemistry	catabolism of pyrimidine base	5.96E-06	2.52E-04
Molecular Transport	transport of irinotecan	5.96E-06	2.52E-04
Cancer, Organismal Injury and Abnormalities, Respiratory Disease	lung cancer	7.79E-06	3.12E-04
Connective Tissue Disorders, Inflammatory Disease, Skeletal and Muscular Disorders	arthritis	1.06E-05	3.78E-04
Cancer, Gastrointestinal Disease, Organismal Injury and Abnormalities	cardiac adenocarcinoma	1.19E-05	3.78E-04
Nucleic Acid Metabolism, Small Molecule Biochemistry	synthesis of dNMP	1.19E-05	3.78E-04
Molecular Transport	transport of SN-38	1.19E-05	3.78E-04
Drug Metabolism, Molecular Transport	transport of cerivastatin	1.19E-05	3.78E-04
DNA Replication, Recombination, and Repair	metabolism of DNA	1.57E-05	4.77E-04

* Ranked by B-H *p*-value.

^Ɨ^ Benjamini-Hochberg multiple testing correction *p*-value.

Table 5 lists the top 25 biological functions discovered by the IPA core analysis of focus genes reported to be associated with mucositis in the literature. These are ranked according to the B-H *p*-values generated, as described in the Methods. The top biological functions identified in the IPA were broadly related to cancer, cell cycle, and DNA replication, recombination, and repair. In this analysis, a smaller *p*-value signifies that the association between the focus genes and the biological functions are non-random. The most significant biological function was related to “cancer, organismal Injury and abnormalities, and skeletal and muscular disorders” with a B-H *p*-value of 1.59^−6^.

### Genetic association between IPA-derived molecules and oral mucositis in head and neck cancer patients

For the genetic association study of oral mucositis, we used the 8 most interconnected molecules (i.e., hubs) derived from the IPA core analysis as listed in Table 3. Among the 8 IPA-derived molecules, P38 MAPK represents a group of genes, including *MAPK1*, *MAPK11*, *MAPK12*, *MAPK13*, and *MAPK14*. Therefore, 8 IPA-derived molecules include 11 candidate genes. After conducting quality control and assessing the availability of SNPs, we had 66 SNPs belonging to the 11 IPA-derived candidate genes that were analyzed for the genetic association study.

Table 6 shows the results for the oral mucositis genetic association study in head and neck cancer patients. The information and results listed in the table include symbols of IPA-derived candidate molecules, cell location, type, number of SNPs belonging to that molecule in the genetic data, gene name, chromosome, and the rs number of the SNP with the most significant *p*-value, the odds ratio (OR) and the *p*-value. The gene RB transcriptional corepressor 1 (*RB1*) showed the highest significance (rs2227311, *p*-value = 0.034, OR = 0.67). The results for all 66 SNPs in the genetic association study are reported in Supporting Information S2 Table.

**Table 6:** Results of the genetic association analysis for oral mucositis in 885 head and neck cancer patients (186 oral mucositis cases and 699 controls), using the IPA-derived hubs (most interconnected molecules) as the candidate molecules.

IPA Symbol^Ɨ^	Location	Type	# of SNPs	Genes	Chr	rs#	OR [95% CI]	*p*-value^ǂ^
*TP53*	Nucleus	transcription regulator	4	*TP53*	17	rs1625895	1.17 [0.83, 1.64]	0.380
CDKN1A**	Nucleus	kinase	3	*CDKN1A*	6	rs3176331	1.11 [0.79, 1.57]	0.545
*CTNNB1*	Nucleus	transcription regulator	4	*CTNNB1*	3	rs3915129	1.03 [0.81, 1.30]	0.805
MYC*	Nucleus	transcription regulator	0	*MYC*	8	-	-	-
*RB1*	Nucleus	transcription regulator	6	*RB1*	13	rs2227311	0.67 [0.46, 0.97]	0.034
P38 MAPK	Cytoplasm	Group	31	*MAPK11*	22	rs742186	0.84 [0.67, 1.06]	0.150
BRCA1**	Nucleus	transcription regulator	13	*BRCA1*	17	rs799917	0.80 [0.63, 1.03]	0.086
*EP300*	Nucleus	transcription regulator	5	*EP300*	22	rs2294976	1.29 [0.85, 1.94]	0.230

^Ɨ^ IPA symbol represents either a gene or a group of genes.

^ǂ^ The most significant *p*-value for a gene or a gene group.

* The genotyping chip does not have SNPs in this gene.

** Focus genes.

CI Confidence interval.

## Discussion

In this study, we performed a comprehensive literature review with the aim of identifying genes that were previously associated with treatment-related mucositis in cancer patients. We then used IPA bioinformatic tools to conduct a comprehensive pathway and network analysis of the genes identified in the literature. From the review of the literature, we found that genes associated with the cell cycle and DNA repair were studied most frequently. Among the focus genes in our study, the genes most commonly assessed for their association with mucositis were *ERCC1*, *XRCC1*, and *MTHFR*. The results of the IPA suggest that the top focus genes, in terms of the number of connections, in mucositis were *CDKN1A* (*p21*) and *BRCA1*; the novel molecules identified through the IPA were *TP53*, *CTNNB1*, *MYC*, *RB1*, P38 MAPK, and *EP300*.

Cancer treatment-induced mucositis is due to DNA damage by antineoplastic therapies. Cancer treatment modalities, in particular radiation therapy, cause oxidative stress by producing reactive oxygen species, which damage DNA and cause cell death [66, 67]. Many of the focus genes identified from the literature review and novel molecules identified from the IPA include regulators and mediators of the DNA damage response and genes directly involved in DNA repair. *ERCC1* is a key component of the nucleotide excision repair complex [68]. *XRCC1* repairs single-strand breaks and is part of the base excision repair pathway [69]. Polymorphisms of DNA repair genes may affect the fate of cells after DNA damage [70]. Common polymorphism in the DNA repair genes might alter an individual’s capacity to repair damaged DNA. It was observed in patients with nasopharyngeal cancers that those with the *XRCC1* 399Arg/Gln genotype were more likely to experience severe acute radiation mucositis [71]. Regulators and mediators of the DNA damage response include *BRCA1*, *RB1* and *TP53*. *RB1* can bind directly to components of the non-homologous end-joining DNA repair machinery [72]. *BRCA1* has important roles in nucleotide excision repair, non-homologous end-joining, and homologous recombinational repair by interacting with components of these DNA repair mechanisms, and by regulating the expression of genes involved in these repair pathways [73]. *BRCA1* interacts with *TP53* and increases *p53*-dependent transcription of *CDKN1A* [74]. *TP53* is a guardian of the genome [75] and is a key mediator of DNA damage response pathways [76]. Mouse models have suggested that the expression of *p53* is increased in irradiated intestinal tissues [77, 78], and higher expression of *p53* has been observed in vitro. *CDKN1A* arrests the cell cycle at the G1 phase to allow adequate time for DNA repair to protect cells against DNA damage and avoid apoptosis [79]. *CTNNB1* is a component of the cadherin cell-cell adhesion complex in epithelia, and its function may be particularly important in the context of mucositis, which involves the breakdown of the mucosa. As a key signaling molecule in the canonical *WNT* signaling pathway, it is also important in cell growth and differentiation during regeneration and repair of the mucosa. *EP300* is a histone acetyltransferase that epigenetically regulates the transcription of genes involved in cell proliferation and differentiation. There is reciprocal regulation between *WNT* and *MYC* [80]. *MYC* participates in the regulation of genomic stability and can regulate the fate of a cell with damaged DNA [81]. The *MAPK* pathway promotes survival and interacts with *MYC* [82]. *MTHFR* is central to folate metabolism and its rs1801131 and rs1801133 SNPs change its enzyme activity [83, 84]. The *MTHFR* C677CT variant with lowered enzyme activity [85] may divert methyl groups from DNA methylation to DNA synthesis [86, 87].

In summary, these genes control cell cycle proliferation, apoptosis, cellular differentiation, DNA repair, and cellular homeostasis [88–91], and their activities may determine the ability of normal mucosa to survive DNA damage or the ability of epithelia to regenerate after tissue death and disruption. Therefore, it is possible that genetic variants in these pathways may also play a role in a cancer patient’s susceptibility to treatment-induced side effects and toxicities, including mucositis.

The present IPA suggests that the P38 MAPK may be involved in the development of mucositis in patients with cancer. Dysregulation of mammalian p38 mitogen-activated protein kinases (*MAPK*s) has been associated with cancer development. Evidence suggests that P38 MAPK activity is critical for normal immune and inflammatory response and that this pathway is a key regulator of proinflammatory cytokines [92]. Previous research shows that P38 MAPK activation is a prerequisite for the production of several cytokines, including interleukin *IL-1*, *IL-8*, *IL-6*, and tumor necrosis factor (*TNF*)-*α* [93]. Dysregulation of *MAPK*s has been associated with cancer development, inflammation, and cancer-related pain [21]. In a previous study of nearly 1400 patients with squamous cell carcinoma of the head and neck (HNSCC), we found that a germline SNP in *MAPK1* showed a significant association with cancer-related pain. Nearly 80% of patients with HNSCC who receive concurrent chemotherapy and radiation will experience mucositis and related symptoms, including severe pain [1].

In addition to their role in pain, cytokines are key players in the processing of and protection from bacterial and viral infections [94]. The last several years have seen a dramatic shift in the understanding of epithelial cell–microbiota interactions, as the commensal microflora are now known to be critical to the health of oral and gastrointestinal epithelial cells and to local and systemic immune function [95–97]. Cancer therapy induces the production of reactive oxygen species that in turn incite an inflammatory response by activating nuclear factor kappa B and amplifying *TNF-α*, which results in the disruption of the epithelial cell barrier and translocation of colonizing bacteria [98]. Activation of the P38 MAPK pathway is a protective reaction against excessive inflammation. Recent studies have suggested that the dominance of certain pathogenic bacterial species can also activate P38 MAPK. Conversely, commensal organisms may suppress proinflammatory cytokines such as *IL-8* via P38 MAPK [99]. Thus, the loss of specific commensal flora may play a key role in mucositis pathophysiology through the P38 MAPK pathway [100, 101]. Further studies are needed to clarify the microbiome–host interactions that may contribute to mucositis development and severity.

Our results also showed that the top canonical pathways associated with the focus genes based on the ratio or *p*-value include glutamate removal from folates, uracil degradation II (reductive), and thymine degradation (Table 4 and S1 Table). Folates are required in a variety of reactions, also known as one-carbon metabolisms, where they act as carriers of one-carbon units in a variety of oxidation states. Tetrahydrofolate polyglutamates are a family of cofactors that carry and chemically activate one-carbon units for biosynthesis [102]. One-carbon metabolism is linked with changes in redox status [103]. Oxidative stress, which has been implicated in the etiology of both cancer and treatment-related toxicities such as mucositis, results from an imbalance in the production of reactive oxygen species, which promotes damage to the cell structure. The uracil degradation II (reductive) pathway is also of particular importance in cancer, because this pathway is responsible for the degradation of CPD0-1327 5-fluoracil, which is an important anticancer drug. Individuals with defects in the enzymes of this pathway have been shown to have adverse response to treatment and be more susceptible to treatment-related toxicities [104, 105].

Retinoate and RXR/RAR signaling, another pathway identified in the IPA, appears to be involved in the risk for mucositis (Table 4). Retinoic acid is an important metabolite of vitamin A in the diet and has been shown to play an important role in cell development and differentiation, as well as in cancer treatment [106]. Hyporetinolemia is associated with severe mucositis in patients who receive hematopoietic stem cell transplants [107], but whether hyporetinolemia contributes to the development of mucositis is not known. Perhaps a clinical trial can be conducted to assess whether the correction of hyporetinolemia by taking vitamin A decreases the incidence and/or severity of mucositis in cancer patients.

We further conducted a genetic association study for oral mucositis based on the IPA-derived candidate molecules (hubs, Table 3). We found that one germline SNP in *RB1* (rs2227311, *p*-value = 0.034, OR = 0.67) showed a protective effect for oral mucositis. This SNP was top ranked (i.e., lowest *p*-value) but not statistically significant after adjusting for multiple comparisons. SNP rs2227311 is located in the 5’-UTR of the *RB1* gene. As discussed above, *RB1* is a regulator and mediator of the DNA damage response and can bind directly to components of the non-homologous end-joining DNA repair machinery. *RB1*’s gene product is a crucial component of the cell cycle control pathways, and loss of its function deprives the pathway of an important mechanism for disrupting cell proliferation through modulation of gene expression [108]. While the importance of rs2227311 has been shown in invasive ovarian cancer [109], to our knowledge, this is the first study showing the potential association of rs2227311 with mucositis in head and neck cancer patients. Additional studies are needed to further explore this association.

Sonis et al. [110] used peripheral blood cells to investigate gene expression changes in five patients being treated for head and neck cancers. As focus genes, they used the genes shown in the analysis to be associated with regimen-related toxicities (e.g., mucositis) and conducted functional analysis using IPA. Our findings are consistent with the results reported in their paper. For example, Sonis et al. showed *p53*, *MYC*, *CTNNB1* and the P38 MAPK signaling pathway as having relationships with mechanisms leading to regimen-related toxicities, which were also identified from the IPA core analysis in the current study (Table 3).

For the purpose of comparison, we also conducted a gene set analysis of the focus genes identified through the literature review, using GeneAnalytics (geneanalytics.genecards.org) [111]. Specifically, we analyzed the focus genes for overrepresentation in SuperPaths, which are collections of individual relevant pathways using nearest neighbor graphing and hierarchical clustering [111–113]. The SuperPath is classified by the adjusted *p*-values (corrected for multiple testing), obtained based on the cumulative binomial distribution: high quality if adjusted *p*-value ≤ 0.0001, medium quality if 0.0001 < *p*-value ≤ 0.05 and low quality if 0.05 < *p*-value. Compared to the SuperPath results, we observed that most of the top canonical pathways identified by IPA (B-H *p*-value < 10^−5^, Table 4) were also discovered by GeneAnalytics with high quality, including uracil degradation, thymine degradation, DNA double-strand break repair and xenobiotic metabolism signaling. We also investigated the diseases associated with the focus genes. A disease is classified as high, medium and low quality by the disease matching score, which was described in detail by Ben-Ari Fuchs et al. [111]. We observed that some of the top diseases identified by IPA (Table 5) were also discovered by GeneAnalytics with high quality, including lung, breast and ovarian cancers.

The present study has limitations. First, because this study is exploratory in nature, further work is needed to characterize the role of individual genes and pathways in the incidence and severity of oral mucositis. Specifically, the association of *RB1* to oral mucositis should be viewed as preliminary and exploratory. Future validation using independent data as well as other cancer sites is required. This association would not be statistically significant if it had been adjusted for multiple comparisons. However, as this is a preliminary and exploratory analysis, such an adjustment is not usually required [114]. In addition, the IPA bioinformatic approach for identifying gene networks has limitations. Edges in IPA are simplified: the IPA designates only a single edge between each pair of molecules in the network, regardless of how many interactions the molecules share. Finally, the sizes of the networks generated by IPA reflect the amount of literature about the focus genes that is available and therefore may be limited by the lack of focus genes identified for mucositis. For the literature review, we focused on human studies and excluded non-human studies, such as those of Bowen et al. [115] and Chang et al. [116], as the biological mechanisms for mucositis in human and animals might not be the same. Nevertheless, this network analysis identified molecules and biological processes that are relevant in mucositis development, thus providing the basis for future studies.

## Supporting information

S1 Fig Figure for each of the molecules with at least 15 connections and the molecules connected to it in the most significant network generated by IPA core analysis for mucositis. (DOCX)

S1 Table Canonical pathways of potential interest discovered by IPA core analysis. (DOCX)

S2 Table Results of 66 SNPs from IPA-derived candidate molecules in the genetic association analysis for oral mucositis in 885 head and neck cancer patients (186 oral mucositis cases and 699 controls). (DOCX)
