# Supplementary material for: Identifying novel genes and biological processes relevant to the development of cancer therapy-induced mucositis: An informative gene network analysis
Source: PLoS One. 2017 Jul 5;12(7):e0180396. doi: 10.1371/journal.pone.0180396 (PMC5498049; doi:10.1371/journal.pone.0180396)

#### Figure S1. Figure for each of the molecules with at least 15 connections and the molecules connected to it in the most significant network generated by IPA core analysis for mucositis. The light blue edges show the connection between the molecules with at least 15 connections and other molecules.

#### (A) *TP53*


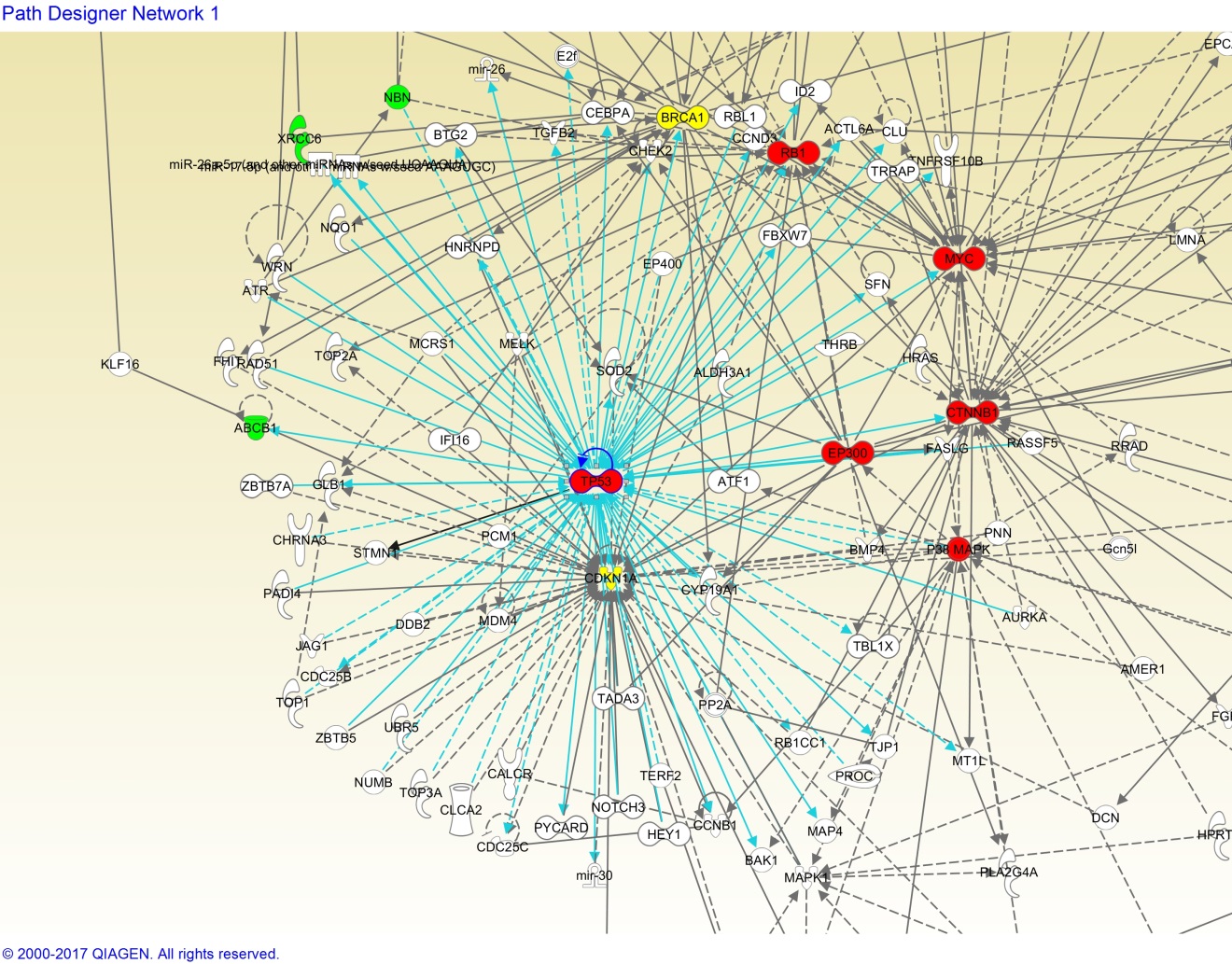


#### (B) *CDKN1A*


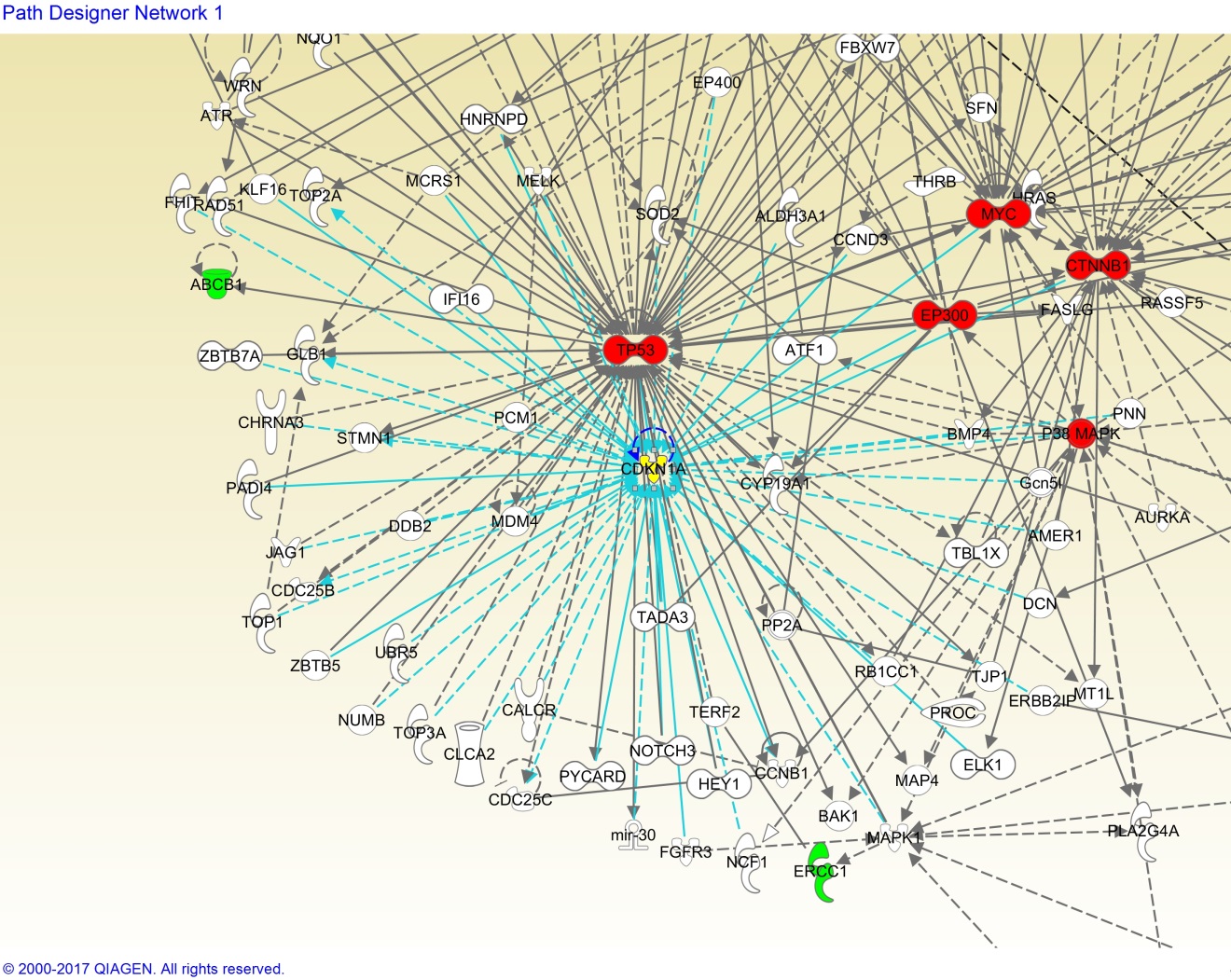


#### (C) *CTNNB1*


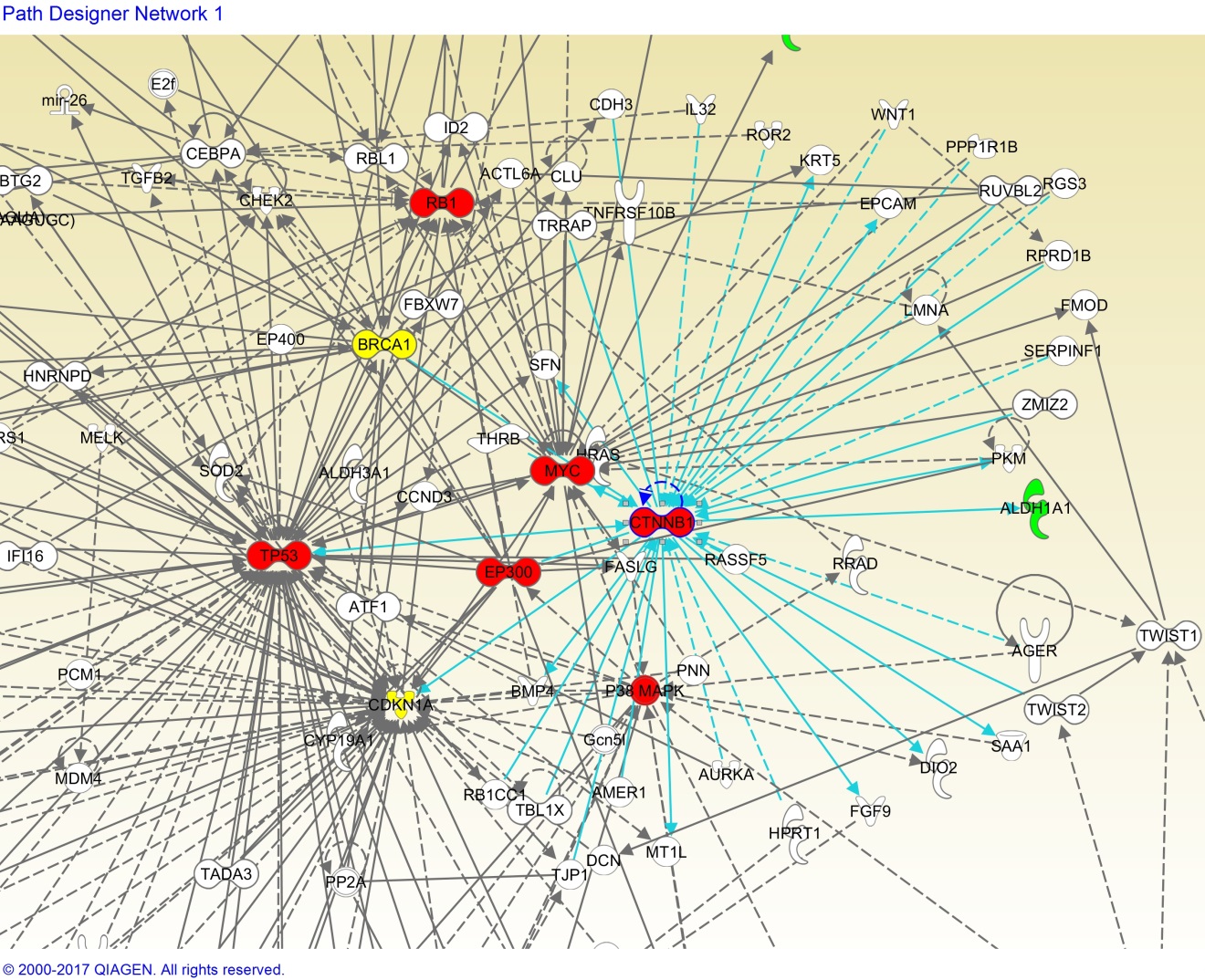


####

#### (D) *MYC*

####
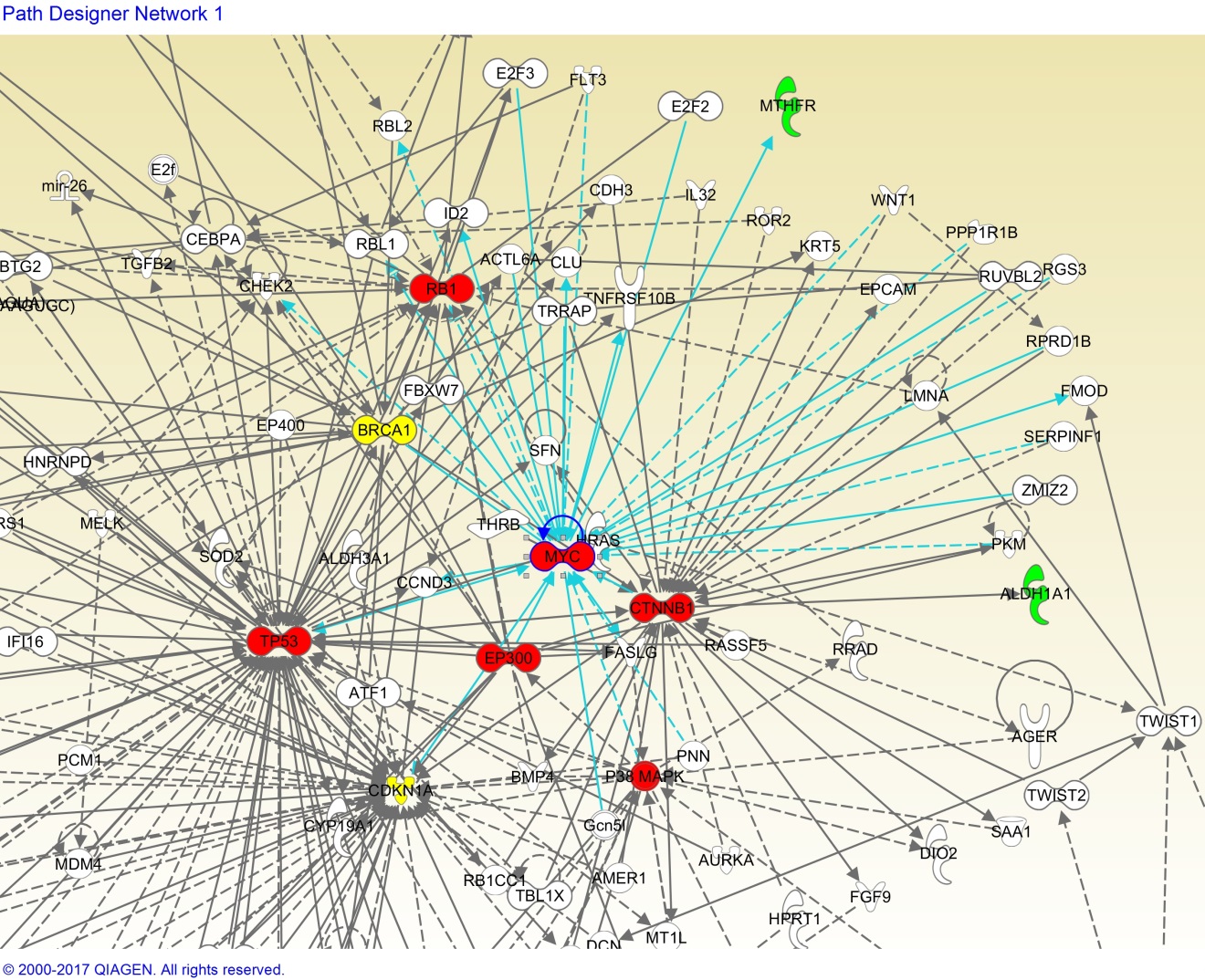


#### (E) *RB1*


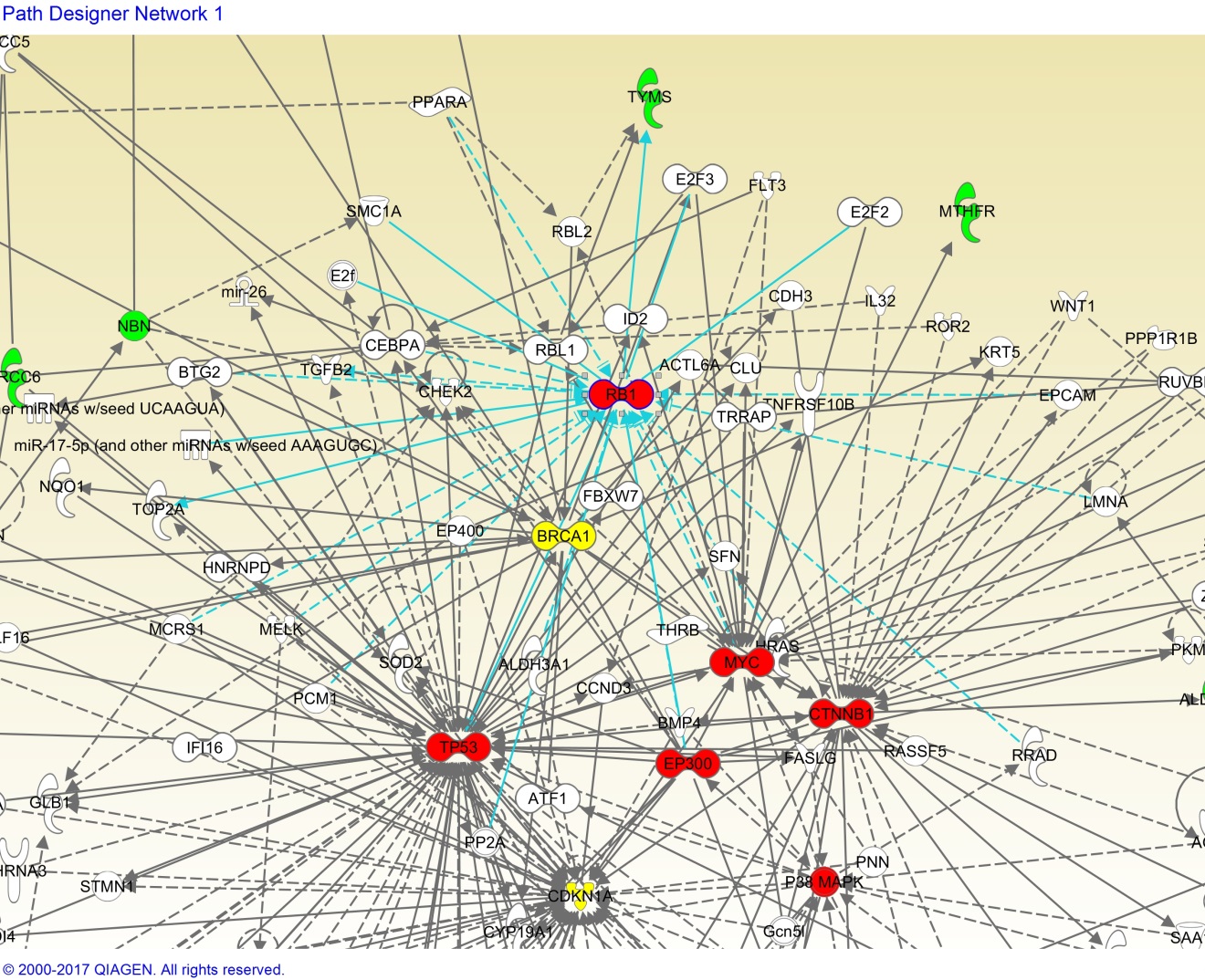


#### (F) P38 MAPK


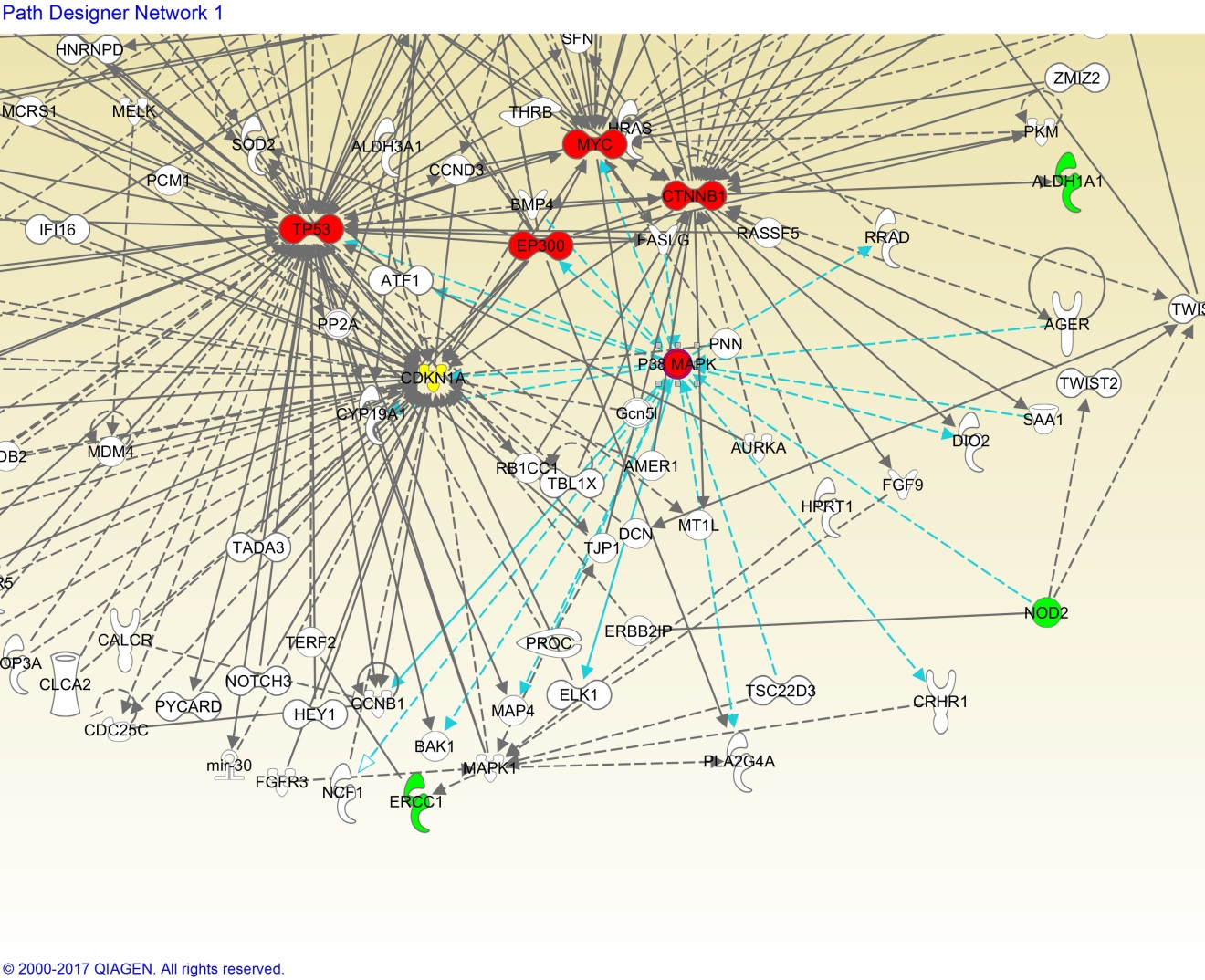


#### (G) *BRCA1*


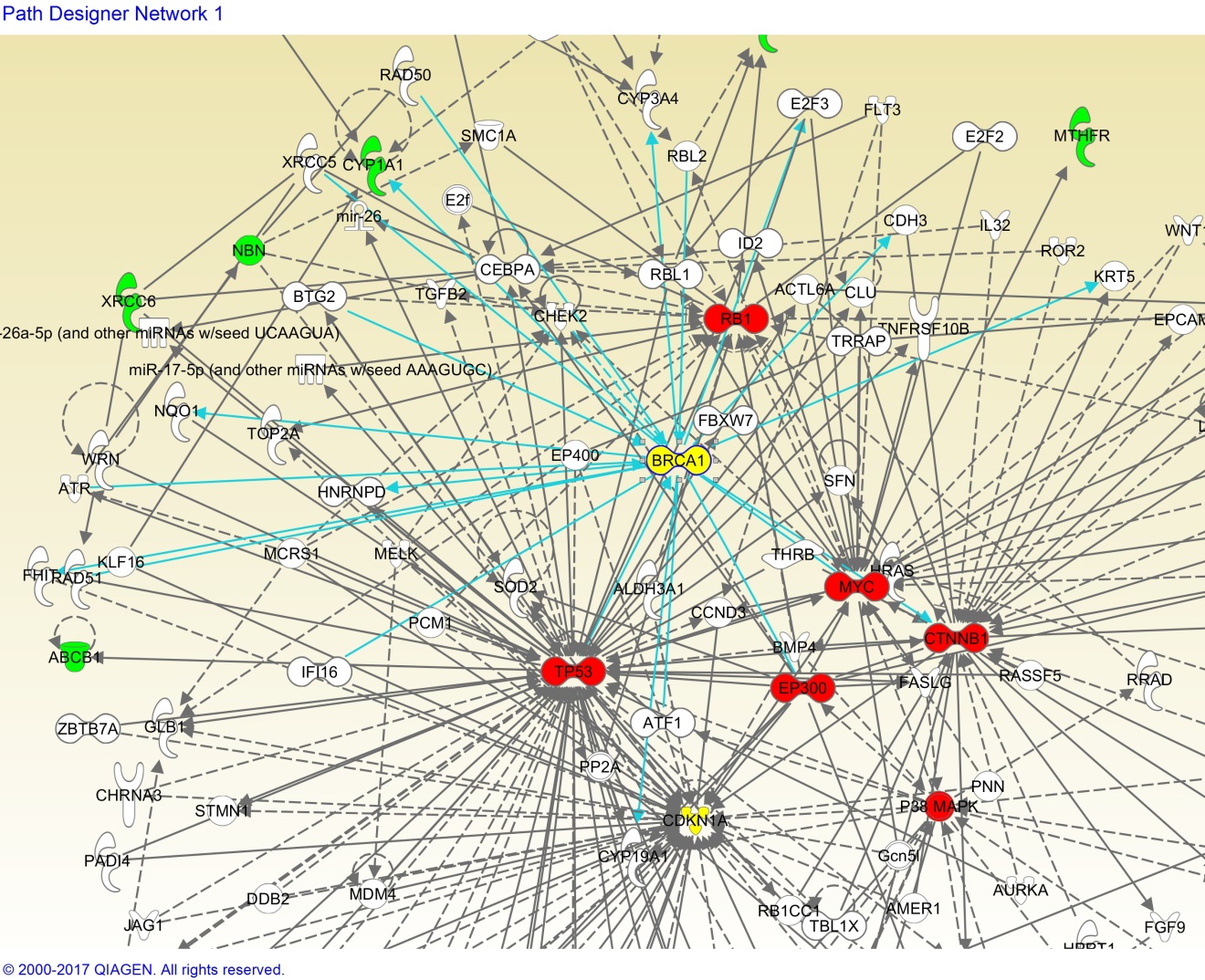


#### (H) *EP300*


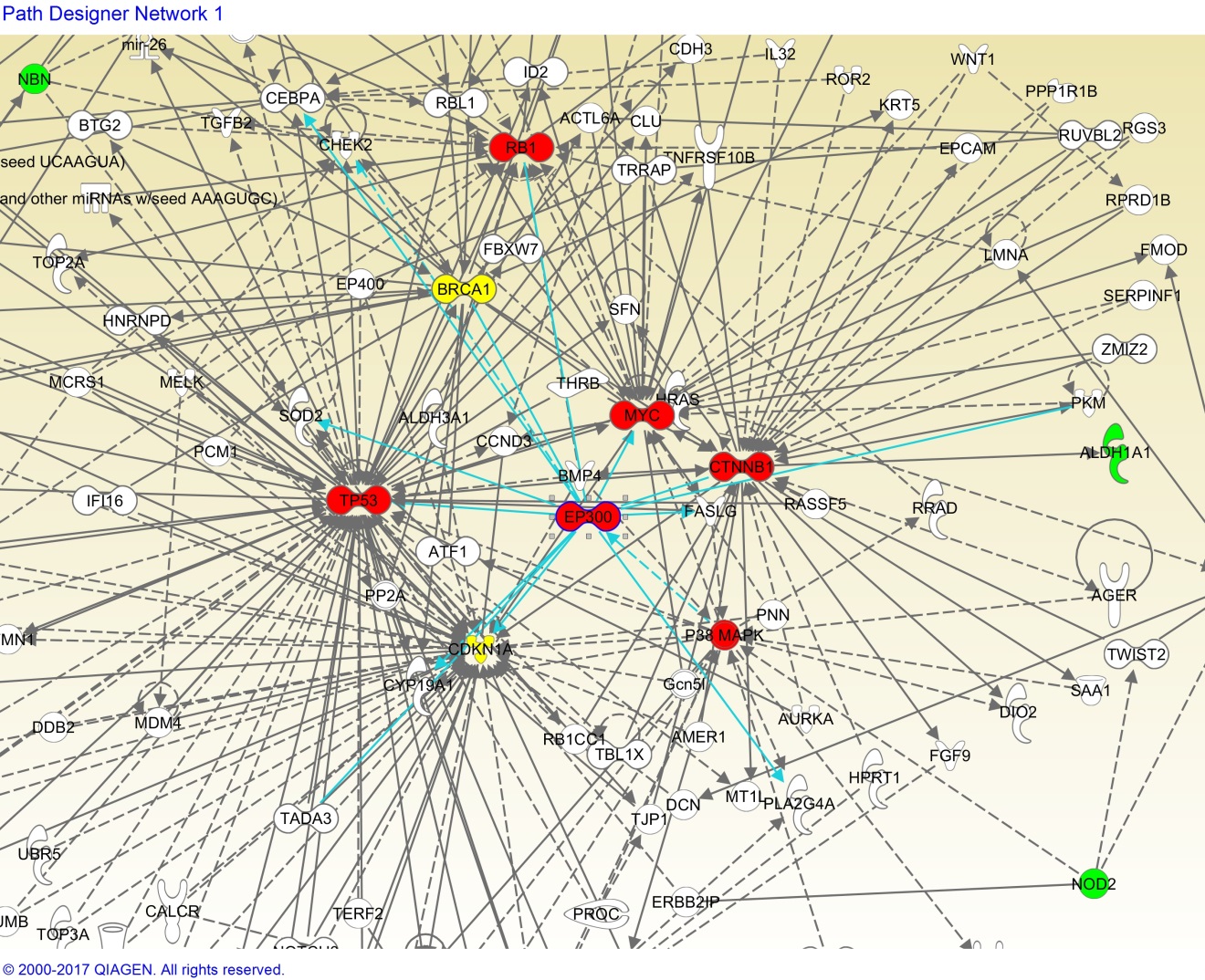

Supplement: S1 Fig — (DOCX) [file pone.0180396.s001.docx]
