# Supplementary material for: Identifying novel genes and biological processes relevant to the development of cancer therapy-induced mucositis: An informative gene network analysis
Source: PLoS One. 2017 Jul 5;12(7):e0180396. doi: 10.1371/journal.pone.0180396 (PMC5498049; doi:10.1371/journal.pone.0180396)
Supplement: S1 Table — (DOCX) [file pone.0180396.s002.docx]

#### Table S1. Canonical Pathways of Potential Interest Discovered by IPA Core Analysis *.

| **Ingenuity Canonical Pathways** | **p-Value** | **B-H**  **p-Value** | **# of molecules in pathway** | **# of focus genes** | **Ratio^ǂ^** |
| --- | --- | --- | --- | --- | --- |
|  |  |  |  |  |  |
| Aryl Hydrocarbon Receptor Signaling | 3.93E-05 | 4.27E-04 | 135 | 4 | 3% |
| Role of CHK Proteins in Cell Cycle Checkpoint Control | 6.61E-05 | 6.27E-04 | 55 | 3 | 5% |
| ATM Signaling | 8.15E-05 | 6.89E-04 | 59 | 3 | 5% |
| DNA Double-Strand Break Repair by Homologous Recombination | 1.79E-04 | 1.29E-03 | 14 | 2 | 14% |
| Role of BRCA1 in DNA Damage Response | 1.87E-04 | 1.29E-03 | 78 | 3 | 4% |
| Telomere Extension by Telomerase | 2.06E-04 | 1.31E-03 | 15 | 2 | 13% |
| GADD45 Signaling | 3.35E-04 | 1.96E-03 | 19 | 2 | 11% |
| Bupropion Degradation | 5.38E-04 | 2.73E-03 | 24 | 2 | 8% |
| Glutathione-mediated Detoxification | 5.38E-04 | 2.73E-03 | 24 | 2 | 8% |
| Acetone Degradation I (to Methylglyoxal) | 6.33E-04 | 3.01E-03 | 26 | 2 | 8% |
| Hereditary Breast Cancer Signaling | 7.66E-04 | 3.42E-03 | 126 | 3 | 2% |
| Retinoate Biosynthesis I | 8.44E-04 | 3.56E-03 | 30 | 2 | 7% |
| Estrogen Biosynthesis | 1.28E-03 | 5.14E-03 | 37 | 2 | 5% |
| Glutamate Removal from Folates | 1.44E-03 | 5.46E-03 | 1 | 1 | 100% |
| NRF2-mediated Oxidative Stress Response | 2.04E-03 | 7.14E-03 | 177 | 3 | 2% |
| Nicotine Degradation III | 2.07E-03 | 7.14E-03 | 47 | 2 | 4% |
| Cell Cycle: G2/M DNA Damage Checkpoint Regulation | 2.24E-03 | 7.39E-03 | 49 | 2 | 4% |
| Melatonin Degradation I | 2.33E-03 | 7.39E-03 | 50 | 2 | 4% |
| Nicotine Degradation II | 2.62E-03 | 7.96E-03 | 53 | 2 | 4% |

* Ranked by B-H p-value.

Ɨ Benjamini-Hochberg multiple testing correction p-value.

ǂ Ratios are calculated by taking the number of focus genes that are included in the canonical pathway divided by the number of the molecules that make up the canonical pathway.
