# Supplementary material for: Identifying novel genes and biological processes relevant to the development of cancer therapy-induced mucositis: An informative gene network analysis
Source: PLoS One. 2017 Jul 5;12(7):e0180396. doi: 10.1371/journal.pone.0180396 (PMC5498049; doi:10.1371/journal.pone.0180396)
Supplement: S2 Table — (DOCX) [file pone.0180396.s003.docx]

#### Table S2. Results of 66 SNPs from IPA-derived candidate molecules in the genetic association analysis for oral mucositis in 885 head and neck cancer patients (186 oral mucositis cases and 699 controls).

| **IPA Symbol^Ɨ^** | **Location** | **Type** | **# of SNPs** | **Genes** | **Chr** | **rs#** | **OR** | ***p*-value** |
| --- | --- | --- | --- | --- | --- | --- | --- | --- |
|  |  |  |  |  |  |  | **[95% CI]** |  |
| *BRCA1** | Nucleus | transcription regulator | 13 | *BRCA1* | 17 | rs12516 | 0.83 [0.64, 1.06] | 0.136 |
|  |  |  |  | *BRCA1* | 17 | rs8176318 | 0.83 [0.65, 1.06] | 0.141 |
|  |  |  |  | *BRCA1* | 17 | rs8176305 | 0.86 [0.56, 1.32] | 0.487 |
|  |  |  |  | *BRCA1* | 17 | rs3737559 | 0.85 [0.56, 1.28] | 0.423 |
|  |  |  |  | *BRCA1* | 17 | rs1060915 | 0.83 [0.64, 1.06] | 0.136 |
|  |  |  |  | *BRCA1* | 17 | rs16942 | 0.81 [0.63, 1.04] | 0.102 |
|  |  |  |  | *BRCA1* | 17 | rs16941 | 0.83 [0.64, 1.06] | 0.135 |
|  |  |  |  | *BRCA1* | 17 | rs799917 | 0.80 [0.63, 1.03] | 0.086 |
|  |  |  |  | *BRCA1* | 17 | rs16940 | 0.81 [0.63, 1.05] | 0.106 |
|  |  |  |  | *BRCA1* | 17 | rs4986850 | 0.87 [0.56, 1.33] | 0.512 |
|  |  |  |  | *BRCA1* | 17 | rs1799950 | 1.11 [0.73, 1.67] | 0.623 |
|  |  |  |  | *BRCA1* | 17 | rs799923 | 1.09 [0.84, 1.42] | 0.523 |
|  |  |  |  | *BRCA1* | 17 | rs799912 | 0.82 [0.64, 1.05] | 0.115 |
| *CDKN1A** | Nucleus | kinase | 3 | *CDKN1A* | 6 | rs2395655 | 1.02 [0.81, 1.29] | 0.866 |
|  |  |  |  | *CDKN1A* | 6 | rs3176320 | 1.05 [0.82, 1.34] | 0.706 |
|  |  |  |  | *CDKN1A* | 6 | rs3176331 | 1.11 [0.79, 1.57] | 0.545 |
| *CTNNB1* | Nucleus | transcription regulator | 4 | *CTNNB1* | 3 | rs3864004 | 1.01 [0.79, 1.27] | 0.958 |
|  |  |  |  | *CTNNB1* | 3 | rs3915129 | 1.03 [0.81, 1.30] | 0.805 |
|  |  |  |  | *CTNNB1* | 3 | rs4135385 | 1.00 [0.75, 1.32] | 0.994 |
|  |  |  |  | *CTNNB1* | 3 | rs2953 | 1.02 [0.81, 1.29] | 0.867 |
| *EP300* | Nucleus | transcription regulator | 5 | *EP300* | 22 | rs9611497 | 1.03 [0.80, 1.31] | 0.844 |
|  |  |  |  | *EP300* | 22 | rs1569858 | 1.00 [0.79, 1.28] | 0.994 |
|  |  |  |  | *EP300* | 22 | rs20551 | 0.93 [0.71, 1.21] | 0.591 |
|  |  |  |  | *EP300* | 22 | rs2294976 | 1.29 [0.85, 1.94] | 0.230 |
|  |  |  |  | *EP300* | 22 | rs2076577 | 1.04 [0.81, 1.32] | 0.772 |
| P38 MAPK | Cytoplasm | Group | 31 | *MAPK1* | 22 | rs9340 | 0.91 [0.72, 1.15] | 0.450 |
|  |  |  |  | *MAPK1* | 22 | rs3810610 | 1.07 [0.86, 1.35] | 0.539 |
|  |  |  |  | *MAPK1* | 22 | rs2298432 | 1.06 [0.83, 1.34] | 0.658 |
|  |  |  |  | *MAPK1* | 22 | rs2006893 | 1.07 [0.85, 1.34] | 0.566 |
|  |  |  |  | *MAPK1* | 22 | rs9607272 | 0.98 [0.75, 1.29] | 0.888 |
|  |  |  |  | *MAPK1* | 22 | rs5999521 | 1.06 [0.85, 1.34] | 0.594 |
|  |  |  |  | *MAPK1* | 22 | rs7290469 | 0.92 [0.73, 1.16] | 0.471 |
|  |  |  |  | *MAPK1* | 22 | rs9610375 | 0.92 [0.73, 1.16] | 0.485 |
|  |  |  |  | *MAPK1* | 22 | rs17759796 | 0.80 [0.58, 1.11] | 0.188 |
|  |  |  |  | *MAPK1* | 22 | rs9610417 | 0.99 [0.75, 1.30] | 0.928 |
|  |  |  |  | *MAPK1* | 22 | rs8136867 | 1.09 [0.87, 1.37] | 0.466 |
|  |  |  |  | *MAPK11* | 22 | rs2076139 | 0.96 [0.73, 1.26] | 0.758 |
|  |  |  |  | *MAPK11* | 22 | rs742186 | 0.84 [0.67, 1.06] | 0.150 |
|  |  |  |  | *MAPK12* | 22 | rs1129880 | 1.06 [0.82, 1.36] | 0.667 |
|  |  |  |  | *MAPK12* | 22 | rs742184 | 1.15 [0.89, 1.48] | 0.278 |
|  |  |  |  | *MAPK13* | 6 | rs1059227 | 0.91 [0.71, 1.17] | 0.464 |
|  |  |  |  | *MAPK13* | 6 | rs2071864 | 1.10 [0.85, 1.42] | 0.471 |
|  |  |  |  | *MAPK13* | 6 | rs2071863 | 0.92 [0.68, 1.25] | 0.593 |
|  |  |  |  | *MAPK13* | 6 | rs2859136 | 0.93 [0.73, 1.19] | 0.569 |
|  |  |  |  | *MAPK14* | 6 | rs851023 | 0.85 [0.61, 1.19] | 0.344 |
|  |  |  |  | *MAPK14* | 6 | rs3804454 | 1.06 [0.80, 1.40] | 0.703 |
|  |  |  |  | *MAPK14* | 6 | rs12199654 | 1.10 [0.67, 1.81] | 0.696 |
|  |  |  |  | *MAPK14* | 6 | rs851019 | 1.01 [0.81, 1.27] | 0.908 |
|  |  |  |  | *MAPK14* | 6 | rs2237093 | 1.05 [0.71, 1.56] | 0.802 |
|  |  |  |  | *MAPK14* | 6 | rs851006 | 0.93 [0.71, 1.21] | 0.575 |
|  |  |  |  | *MAPK14* | 6 | rs2815806 | 0.85 [0.61, 1.19] | 0.346 |
|  |  |  |  | *MAPK14* | 6 | rs7760405 | 1.05 [0.73, 1.50] | 0.811 |
|  |  |  |  | *MAPK14* | 6 | rs9470219 | 0.98 [0.78, 1.23] | 0.864 |
|  |  |  |  | *MAPK14* | 6 | rs6457878 | 1.00 [0.70, 1.44] | 0.987 |
|  |  |  |  | *MAPK14* | 6 | rs3804452 | 1.00 [0.69, 1.45] | 0.982 |
|  |  |  |  | *MAPK14* | 6 | rs3804451 | 1.01 [0.70, 1.46] | 0.949 |
| *RB1* | Nucleus | transcription regulator | 6 | *RB1* | 13 | rs4151467 | 0.93 [0.55, 1.58] | 0.794 |
|  |  |  |  | *RB1* | 13 | rs4151510 | 0.67 [0.46, 0.98] | 0.037 |
|  |  |  |  | *RB1* | 13 | rs198607 | 1.08 [0.83, 1.39] | 0.583 |
|  |  |  |  | *RB1* | 13 | rs198604 | 1.03 [0.79, 1.35] | 0.823 |
|  |  |  |  | *RB1* | 13 | rs2227311 | 0.67 [0.46, 0.97] | 0.034 |
|  |  |  |  | *RB1* | 13 | rs2854344 | 1.33 [0.85, 2.08] | 0.206 |
| *TP53* | Nucleus | transcription regulator | 4 | *TP53* | 17 | rs1625895 | 1.17 [0.83, 1.64] | 0.380 |
|  |  |  |  | *TP53* | 17 | rs1042522 | 0.97 [0.74, 1.28] | 0.837 |
|  |  |  |  | *TP53* | 17 | rs8079544 | 1.01 [0.58, 1.76] | 0.982 |
|  |  |  |  | *TP53* | 17 | rs11652704 | 0.93 [0.65, 1.33] | 0.685 |

Ɨ IPA symbol represents either a gene or a group of genes.

* Focus genes.

CI Confidence interval.
